# Hierarchical YOLO-SAM: A Scalable Pipeline for Automated Segmentation and Morphometric Tracking of Coral Recruits in Time-Series Microscopy

**DOI:** 10.3390/s26082291

**Published:** 2026-04-08

**Authors:** Richard S. Zhao, Cuixian Chen, Meg Van Horn, Nicole D. Fogarty

**Affiliations:** 1 Department of Computer Science, University of North Carolina at Chapel Hill, Chapel Hill, NC 27599, USA; 2 Department of Mathematics and Statistics, University of North Carolina Wilmington, Wilmington, NC 28403, USA; 3 Department of Biology and Marine Biology, Center for Marine Science, University of North Carolina Wilmington, Wilmington, NC 28403, USA; meg.vanhorn98@gmail.com (M.V.H.); fogartyn@uncw.edu (N.D.F.)

**Keywords:** YOLO, segment anything model (SAM), object detection, instance segmentation, microscopy imagery, growth measurement, coral recruit, marine ecology

## Abstract

Coral reef ecosystems are declining rapidly due to climate change, disease, and anthropogenic stressors, driving the expansion of land-based coral propagation for reef restoration. A major bottleneck in these efforts is the manual measurement of coral recruit tissue area from microscopy images, which requires 2–7 min per image and limits scalability. We present a hierarchical deep learning pipeline that automates this measurement by integrating YOLO-based detection with Segment Anything Model (SAM) segmentation. YOLO localizes recruits and classifies them by developmental stage; stage-specific fine-tuned SAM models then segment live tissue using bounding box and background point prompts to suppress segmentation leakage and improve boundary precision. Surface area is computed directly from the segmented masks using pixel size extracted from image metadata. The pipeline reduces processing time to approximately 3–5 s per image—a 24–140× speedup over manual tracing. Evaluated on 3668 microscopy images from two national coral research facilities, the system achieves a mean IoU exceeding 95% and an auto-acceptance rate (AAR) of 71.51%, where predicted-to-ground-truth area ratios fall within a ±5% tolerance of expert annotation, substantially reducing manual workload while maintaining measurement reliability across species, developmental stages, and imaging conditions. This workflow addresses a critical bottleneck in restoration research and demonstrates the broader applicability of AI-based image analysis in marine ecology.

## 1. Introduction

Coral reefs are among the most biodiverse ecosystems on Earth, providing multiple ecosystem services and playing a key role in global economies [1,2,3]. However, global coral cover has declined by approximately 50% since 1957 [4,5]. Climate change is the main driver of this decline, with increased sea surface temperatures causing global coral bleaching events that are increasing in frequency and severity [6,7] Although local management efforts exist, they are largely ineffective against such global stressors, making restoration and intervention strategies an essential pillar of reef conservation [8].

Coral recruitment, the process in which coral larvae settle and metamorphose on substrates or existing reefs, is vital to restoration efforts [9]. Ex situ spawning facilities simulate temperature and light cycles to spawn corals for research and restoration efforts. Some of these facilities are still in their infancy, and researchers are working to understand how to optimize coral production by understanding the factors (e.g., substrate material, water flow, lighting, nutrition) that affect recruit health and growth [10,11,12,13]. These facilities measure recruit growth over time by manually tracing the polyp in microscopy images and using software to calculate the surface area [14,15]. However, manual tracing is slow (2 to 7 min per image) and labor intensive, limiting its scalability for large-scale restoration trials [16,17]. Traditional machine learning methods have been used to segment coral tissue, but struggle to generalize between datasets with varying lighting conditions, substrate types, species, and coral morphologies [18]. Other automated systems, often designed for reef surveys or remote sensing, lack the resolution and accuracy for millimeter-scale analysis of juvenile coral [18,19,20,21,22,23,24].

To overcome these challenges, we propose a hybrid framework that integrates YOLO (You Only Look Once) for efficient region-of-interest detection with SAM (Segment Anything Model). Note that YOLO and SAM are used throughout as general terms for their respective model families, unless otherwise specified. By unifying high-throughput detection with precise, annotation-free pixel-level segmentation, this approach eliminates the need for costly manual labeling. this approach significantly reduces the need for costly manual labeling. Unlike traditional machine learning methods, deep learning models automatically extract hierarchical features from data [25,26], enabling robust generalization across complex imaging conditions—a quality that has proven effective in medical imaging tasks requiring high precision in biological structures [27].

We further contribute three targeted improvements to enhance segmentation accuracy and biological relevance:

- *Stage-specific fine-tuning.* Two dedicated SAM models are trained separately for early- and late-stage coral recruit development, directly addressing the distinct morphological and imaging challenges of each developmental phase.
- *Prompt engineering with negative prompts.* Background points are incorporated as negative prompts alongside standard bounding box inputs, constraining mask expansion—a critical refinement for early-stage recruits where boundary ambiguity is greatest.
- *End-to-end evaluation.* We present a comprehensive assessment of the full pipeline, demonstrating that automated, high-throughput analysis can match or exceed expert annotation accuracy while reducing processing time by one to two orders of magnitude (24–140×).

Together, these contributions establish a reproducible and scalable framework for coral recruit monitoring across large datasets, offering a practical path toward automating reef assessment at ecologically meaningful scales.

## 2. Materials

### 2.1. Data Collection and Background

This study used coral recruit images from two research facilities: the Coral REEF Laboratory at the University of North Carolina Wilmington (UNCW) and the Mote Marine Laboratory and Aquarium (Mote). Although there are slight differences in equipment and procedures employed by the two laboratories, both use high-resolution microscopes to capture juvenile coral recruits that are difficult to detect with the naked eye. At UNCW, images were taken using an Olympus LC30 camera (Olympus Corporation, Tokyo, Japan). mounted on an SZ61 stereo microscope (Olympus Corporation, Tokyo, Japan). This was used in tandem with the cellSens imaging software (cellSens Entry version, Olympus Corporation, Tokyo, Japan), allowing for embedded pixel-to-micrometer conversion metadata in the final TIFF image.

Coral recruit growth images from three projects were analyzed. Table 1 showed that the datasets span three cohorts collected at two laboratories over two years and include four coral species. UNCW datasets (2023–2024) comprise of *Pseudodiploria clivosa* (PCLI) and *Montastraea cavernosa* (MCAV) recruits photographed at seven time points at approximately 4-week intervals, with image counts declining from ∼400 to ∼200 as recruits died or were removed. The Mote dataset (2024) includes *Colpophyllia natans* (CNAT) and *Pseudodiploria strigosa* (PSTR) recruits photographed at four time points at approximately 8-week intervals, with image counts declining from ∼600 to ∼400.

Approximately one-third of the images were used for YOLO-SAM model development and split into training, validation, and test sets with a ratio of (70:20:10). Performance was evaluated systematically using the two most recent datasets (UNCW 2024 and Mote 2024) with a total of 3668 images. Together, these datasets capture variability in species composition, temporal sampling, and laboratory imaging workflows, enabling assessment of the deep learning pipeline’s robustness and cross-laboratory generalizability.

**Figure 1:**
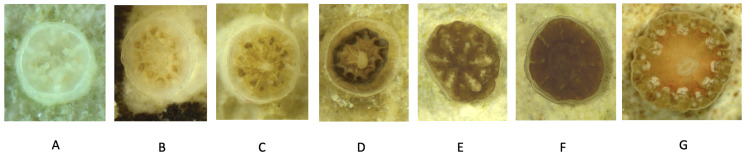
Qualitative estimates of zooxanthellae density from Stage (**A**–**G**), across Weeks 0 to 28 post-fertilization.

**Figure 2:**
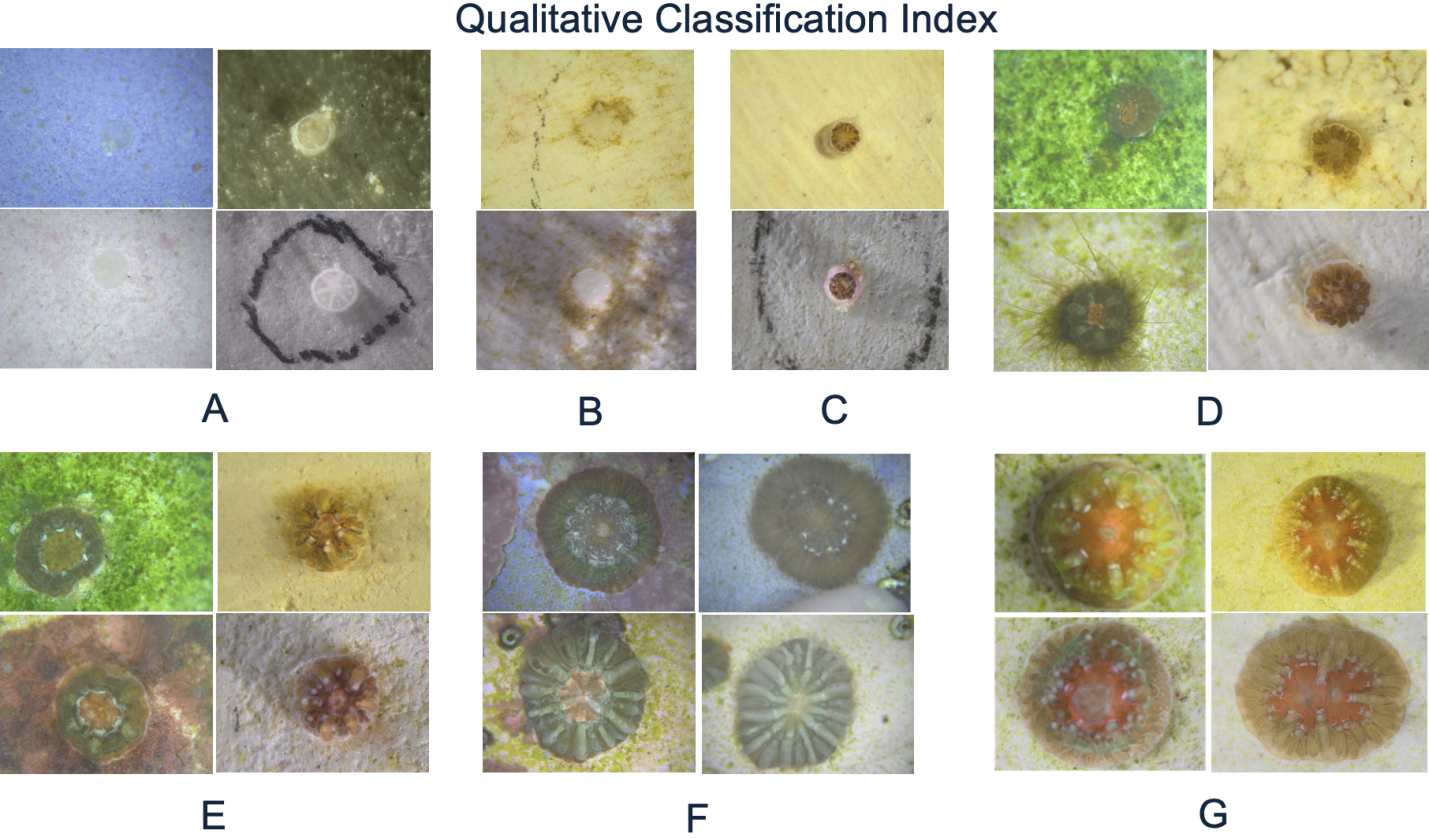
Example images from each stage (**A**–**G**), highlighting tissue coloration, maturity, and growth progression.

With this data, a qualitative classification system based on tissue coloration was provided. The images of coral recruits were assigned to categories A-G according to tissue coloration, growth maturity, and visual development (see Figure 1). This was necessary since coral growth rates varied between tanks, making comparisons by time point less effective. This qualitative classification allowed the pipeline to be evaluated based on visual distinction, rather than just by weeks. Furthermore, having a qualitative classification system made it easier to define methodologies to annotate images. Specifically, stage A–C is defined as early-stage, while stage D–G is defined as late-stage. Moreover, Figure 2 shows representative examples of each qualitative classification from different species/laboratories, lighting conditions, and backgrounds.

### 2.2. Image Preparation and Data Augmentation

The training dataset was restricted to microscopy images containing a single, isolated coral recruit, consistent with the study’s focus on individual-level detection, segmentation, and live tissue measurement. Images were excluded if they contained overlapping or adjacent recruits, physical damage, septal overgrowth, or imaging artifacts—specifically overexposure, underexposure, blur, or uneven illumination—identified through systematic quality screening prior to annotation. Although this single-recruit constraint reflects the scope of available ground-truth annotations rather than an architectural limitation, the framework is extensible to multi-recruit images, requiring only annotated training data with individual bounding boxes and segmentation masks per recruit.

Data augmentation was applied to the training images to improve generalization and prevent overfitting, following best practices in deep learning image analysis [28,29]. Training data was augmented threefold using rotations, grayscale conversion, and brightness and saturation adjustments, applied during both YOLO training and SAM fine-tuning to improve robustness without increasing model complexity or inference cost. Geometric augmentations included horizontal and vertical flips, random rotations (±15°), 90° rotations, and random cropping (<20% zoom). Color and noise augmentations comprised grayscale conversion (15% of images), hue shifts (±10°), saturation and brightness adjustments (±20%), exposure changes (±15%), Gaussian blurring (<2.5 pixels), and random noise injection (0.1% of pixels). Images were resized to meet the requirements of each model: 640 × 640 pixels for YOLO, where coarse features suffice for recruit detection, and 1024 × 1024 pixels for SAM, where higher resolution is needed for precise tissue segmentation.

### 2.3. Coral Recruit Annotation

**Figure 3:**
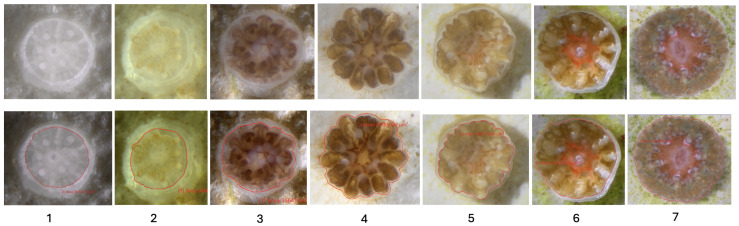
Representative coral recruits across post-settlement developmental stages. Top row shows raw microscopy images and bottom row shows corresponding manual tissue annotations in cellSens (red). Stages progress from early recruits without symbionts (**1**), to symbiont-colonized and increasingly developed recruits with varying degrees of skeletal cup exposure (**2**–**6**), culminating in large recruits with complete tissue coverage of the skeletal cup (**7**).

Ground-truth surface areas were generated for the two recent growth projects (UNCW 2024 and Mote 2024) to complete the full AI system evaluation. Annotation guidelines provided by experts from the UNCW Coral REEF Laboratory informed a standardized polygon tracing scheme, implemented in cellSens, that delineated only the live tissue (polyp) of each coral recruit while excluding the corallite cup (Figure 3).

## 3. Methods

### 3.1. Hierarchical Developmental-Stage Architecture

In Figure 3, the coral microscopy images exhibit pronounced, developmentally driven heterogeneity in tissue morphology, symbiont pigmentation, and tissue–skeleton contrast across post-settlement stages. Early-stage recruits showed thin, weakly pigmented tissue confined to the skeletal cup and diffuse boundaries, and late-stage recruits displayed thicker, continuous tissue with clearer skeletal separation.

In our preliminary studies, initial fine-tuning of a single SAM model using a mixed dataset of early-stage and late-stage coral recruits resulted in degraded segmentation performance, particularly for early-stage corals, where segmentation masks expanded beyond the biologically relevant tissue region. This behavior was attributed to stage-dependent boundary semantics: early-stage coral recruits require segmentation confined to the upper tissue layer, whereas late-stage corals require outward expansion to capture thicker tissue and skeletal structure.

To address these challenges, we proposed a hierarchical architecture that explicitly accounts for the coral developmental stage. First, a YOLO-based detector was trained with an additional class label to classify coral recruits into developmental categories (e.g., early- vs. late-stage). Secondly, two stage-specific SAM models were fine-tuned independently using only images from their corresponding developmental stage, ensuring consistent boundary supervision within each model. During inference, each image is first classified by developmental stage, and then the corresponding stage-specific SAM model is applied for segmentation. This hierarchical approach reduces conflicting supervision during fine-tuning and produces segmentation masks that are more consistent with the biological morphology of coral recruits throughout developmental stages.

**Figure 4:**
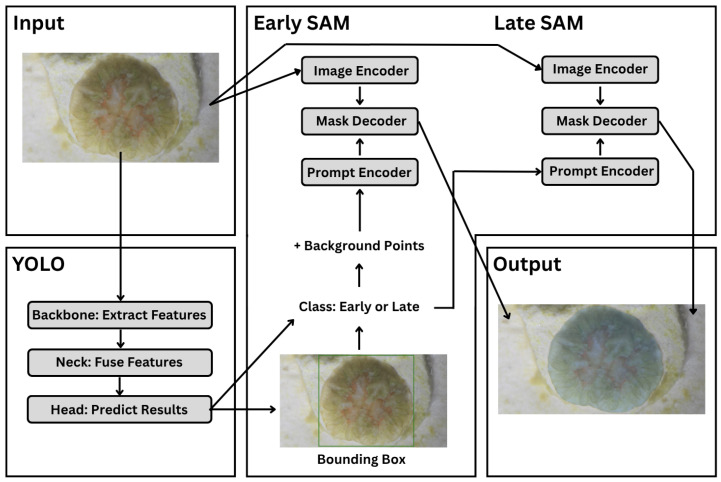
Hierarchical architecture of the proposed YOLO–SAM framework with stage-specific prompt strategies: YOLO performs object detection and stage classification. Early SAM uses bounding-box prompts with four additional background points as negative prompts, while Late SAM uses bounding-box prompts only.

The proposed architecture (Figure 4) consists of an object detection model based on YOLO to detect and classify coral recruits, and two SAM-based segmentation models that delineate coral tissue for early or late coral images. The YOLO model performs detection to generate a bounding-box prompt that indicates regions of interest and classification to identify the coral recruit as early- or late-stage. This allows for the correct SAM to be chosen, while also guiding the SAM to focus on regions of interest for more detailed segmentation. If the coral recruit is classified as early-stage, further prompt engineering is used by adding negative prompts at each side of the bounding box to greater restrict the SAM.

### 3.2. YOLO

YOLO (You Only Look Once) is a single-stage object detector that performs localization and classification in a single forward pass. Its architecture comprises a convolutional backbone for hierarchical feature extraction, a neck for multi-scale feature aggregation, and a detection head that outputs bounding box coordinates and class probabilities. This design balances inference speed with detection accuracy across object sizes, making it well suited for high-volume image processing workflows. In this study, the YOLO module detects and classifies coral recruits in microscopy images, where multi-scale detection is particularly advantageous for small or low-contrast targets [30]. YOLO generates bounding boxes used as prompts for SAM and classifies recruits as early- or late-stage, enabling automatic selection of the appropriate SAM model. Unlike SAM, YOLO is pretrained on general object categories (COCO) and requires fine-tuning on domain-specific data for reliable coral recruit detection.

The YOLO module was iteratively refined to improve detection, particularly for early-stage recruits with poorly defined tissue boundaries. Initial YOLOv8 [31] trials produced occasional bounding box misalignments that propagated localization errors to downstream segmentation. Upgrading to YOLOv11 [32] improved robustness under high background clutter and low-contrast conditions. Grayscale preprocessing was evaluated to reduce color dependence but yielded no improvement over RGB, confirming that chromatic cues are biologically informative. For the final model, YOLOv11 was fine-tuned on a labeled dataset of coral recruit microscopy images from two research laboratories, augmented with images from the Mote growth project to improve diversity across growth stages and imaging conditions. Recruits were annotated with bounding boxes and classified as early- or late-stage. The dataset was split into 70% training, 20% validation, and 10% testing, using default Ultralytics hyperparameters except for 50 epochs and a batch size of 4 due to GPU memory constraints, with automatic mixed precision (AMP) enabled. Given the high detection accuracy achieved, recruit localization was treated as a resolved component and held fixed for subsequent pipeline development.

### 3.3. SAMs

SAM’s encoder–decoder design processes images and prompts (e.g., bounding boxes) independently before combining them to produce binary segmentation masks, enabling strong zero-shot generalization through pretraining on 11 million images [33]. When a specific version is referenced, SAM ViT-H denotes the original model [33] and SAM2-Hiera-Large denotes the second-generation model [34]; the two differ principally in backbone architecture, with SAM ViT-H using a plain Vision Transformer encoder and SAM2-Hiera-Large adopting a hierarchical Hiera encoder with a larger attention-head count and reduced checkpoint size.

In our pipeline, YOLO-derived bounding boxes and negative prompts guide SAM to segment live coral tissue from surrounding corallite, skeleton, and substrate. Although SAM generalizes well in zero-shot settings, initial performance on early-stage recruits was inconsistent due to indistinct tissue boundaries and low contrast. To address this, we fine-tuned two separate SAM models—one for each developmental stage—using manually annotated segmentation masks. Early-stage fine-tuning focused on reducing over-segmentation into the corallite cup, while late-stage fine-tuning improved boundary delineation along the tissue edge. Both models were trained using YOLO-derived bounding boxes as prompts and manual segmentation masks as ground truth, for 40 epochs with a batch size of 4, a learning rate of $1\times 10^{-5}$, and a weight decay of $4\times 10^{-5}$ to preserve pretrained weights during adaptation. These stage-specific models replaced the default SAM checkpoint in the final pipeline. All model development and training were conducted on cloud-based GPU resources (Google Colab). Both stage-specific SAM2-Hiera-Large models were trained for up to 40 epochs with early stopping; validation loss plateaued at approximately 18 and 25 epochs for early-stage and late-stage SAM models, respectively. Fine-tuning was performed on NVIDIA A100 GPUs (∼7 min per epoch; ∼2–3 h per model). In contrast, training on NVIDIA T4 GPUs required ∼20 min per epoch. All inference experiments were conducted on NVIDIA T4 GPUs.

**Figure 5:**
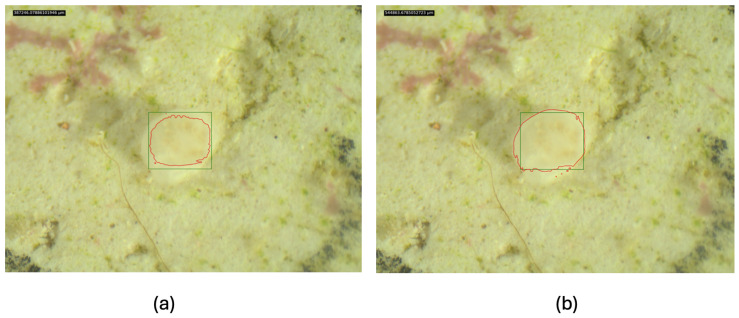
Effect of background-point prompts on early-stage SAM2 segmentation: (**a**) with four background (negative) points placed at the midpoints of the bounding-box edges and (**b**) without background-point prompts. Green: YOLO bounding box; red: SAM2 segmentation contour.

Segmentation performance was further improved through prompt engineering. Beyond bounding boxes, we added four background points at the midpoint of each bounding-box edge as negative prompts to suppress mask expansion into non-tissue regions—particularly the corallite cup and surrounding substrate in early-stage recruits. Each bounding box is defined by its corner coordinates $\left(x_{\min},y_{\min}\right)$ and $\left(x_{\max},y_{\max}\right)$, recorded as absolute pixel coordinates alongside the source image dimensions; four background points are placed at the midpoints of each edge—$\left(x_{c},y_{\min}\right)$, $\left(x_{c},y_{\max}\right)$, $\left(x_{\min},y_{c}\right)$, and $\left(x_{\max},y_{c}\right)$—where $x_{c}=\frac{x_{\min}+x_{\max}}{2}$ and $y_{c}=\frac{y_{\min}+y_{\max}}{2}$. As shown in Figure 5, adding four background (negative) points at the midpoints of the bounding-box edges (Figure 5a) reduced mask leakage and improved tissue boundary delineation under low-contrast conditions where tissue and cup are difficult to distinguish, compared to bounding-box prompts alone (Figure 5b). This was especially critical given that small boundary errors can propagate into large relative errors in downstream area estimates.

We evaluated three SAM variants—SAM ViT-H [33], SAM2-Hiera-Base-Plus, and SAM2-Hiera-Large [34]—selecting SAM2-Hiera-Large for its greater number of attention heads and superior negative prompt utilization. SAM2’s Hiera encoder achieves comparable representational capacity to ViT-H with fewer parameters, and prior evaluations confirm it matches or exceeds SAM ViT-H performance under proper calibration. Combined with fine-tuning and stage-specific model selection, these strategies adapted SAM2 into a high-precision segmentation module for coral morphology. Hereafter, SAM2 refers specifically to SAM2-Hiera-Large unless otherwise stated. Stage-specific SAM2 configurations are summarized in Table 2.

### 3.4. Performance Evaluation Metrics

Pipeline performance was evaluated at two levels. Standard machine learning metrics assessed YOLO and SAM individually during training. End-to-end evaluation then compared three configurations—prototype, optimized, and class-specific model switching in an ablation study—to quantify the effects of prompt design, fine-tuning, and stage-based model selection on auto-acceptance rate (AAR) and measurement accuracy.

#### 3.4.1. Component-Level Evaluation

During fine-tuning, YOLO and SAM were evaluated on held-out validation sets to guide model selection. YOLO was assessed using precision, recall, and mean average precision at IoU threshold 0.5 (mAP@50), capturing localization accuracy and detection reliability. SAM was evaluated using Intersection over Union (IoU) between predicted and manually traced ground-truth masks, which penalizes both over- and under-segmentation and is sensitive to boundary misalignment—critical for area estimation in growth analysis. Loss and performance curves were tracked across epochs to monitor learning stability. Additionally, both models were evaluated on a held-out test set to confirm generalization and rule out overfitting or data leakage.

#### 3.4.2. Pipeline-Level Evaluation

Three pipeline configurations were evaluated to quantify developmental improvements. The prototype used YOLOv8, SAM ViT-H, box-only prompts, and no fine-tuning, serving as a baseline control. The final configuration used YOLOv11, SAM2-Hiera-Large, bounding box and background prompts, and stage-specific fine-tuned SAM models, representing the final optimized pipeline. A third class-specific configuration inverted the model–stage assignments to validate that each fine-tuned SAM performed best on its intended recruit stage, confirming the dual-branch design. Configuration differences are summarized in Table 3. All configurations were evaluated on the UNCW 2024 and Mote 2024 coral growth datasets. Unlike component-level evaluation, which targeted segmentation and detection accuracy (IoU, mAP@50), pipeline-level evaluation assessed measurement accuracy—how closely predicted recruit areas matched ground-truth measurements—using multiple statistical indicators at each time point.

##### Surface Area Quantification

The real-world surface area ($A_{\mathrm{real}}$) was computed directly from the segmented masks using pixel size extracted from the image metadata, as follows:

(1) $$A_{\mathrm{real}}=A_{\mathrm{px}}\times s^{2},$$

where $A_{\mathrm{px}}$ denotes the number of pixels within the segmented mask and *s* the pixel size (real-world length per pixel). When the pixel size is provided in micrometers per pixel (μm/pixel), the surface area is obtained in square micrometers (μm^2^).

##### Area Ratio (AR)

The area ratio quantifies systematic bias in a predicted area relative to ground truth:

(2) $$AR_{i}=\frac{A_{i}^{\mathrm{pred}}}{A_{i}^{\mathrm{true}}},\;i=1,\ldots ,N,$$

where $A_{i}^{\mathrm{pred}}$ and $A_{i}^{\mathrm{true}}$ are the predicted and manually traced areas for the *i*-th image, and *N* is the total number of images at a given time point. AR=1 indicates perfect agreement; AR>1 and AR<1 indicate over- and underestimation, respectively.

##### Auto-Acceptance Rate (AAR)

The AAR is the proportion of predictions within 5% of the ground truth area:

(3) $$\mathrm{AAR}=\frac{1}{N}\sum_{i=1}^{N}1\left(\left|AR_{i}-1\right|<0.05\right)\times 100,$$

where 1(·) is an indicator function equal to one when $AR_{i}\in \left[0.95,1.05\right]$ and zero otherwise. Metrics were computed at each time point across both datasets to capture temporal variability.

## 4. Results

To validate individual components and fine-tuning effects, we evaluated detection and segmentation modules prior to full pipeline testing, ensuring each module achieved the precision required for accurate coral recruit measurement.

### 4.1. Object Detection Performance (YOLO)

Table 4 reports YOLOv11 detection performance on the held-out test set. High precision, recall, and mAP@50 across both stages indicate robust recruit localization suitable for downstream segmentation. The lower mAP@50-95 for early-stage recruits (0.873 vs. 0.948) reflects their diffuse tissue margins and reduced contrast against the skeleton, which makes precise bounding-box alignment more challenging at stricter IoU thresholds.

**Figure 6:**
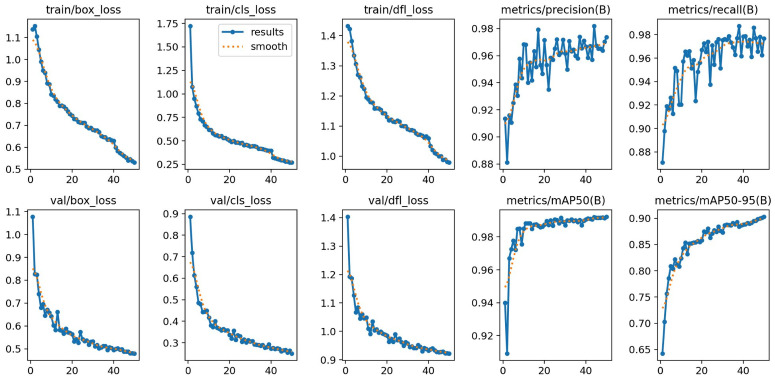
YOLOv11 training and validation performance across epochs.

Training curves (Figure 6) show stable convergence over 50 epochs without overfitting, with validation metrics closely tracking training performance—indicating effective generalization across species, lighting conditions, and substrate types in the dataset.

### 4.2. Segmentation Performance (SAM)

Both stage-specific SAM models achieved high segmentation accuracy (Table 5). The late-stage model reached an IoU of 0.9760 and the early-stage model 0.9598. The early-stage model achieved a lower final loss (0.0025 vs. 0.0100) but slightly lower IoU, consistent with the greater ambiguity at early-stage tissue boundaries where live tissue and corallite cup are difficult to distinguish. The late-stage model’s higher loss but superior IoU reflects successful adaptation to more complex but better-defined tissue morphologies.

**Figure 7:**
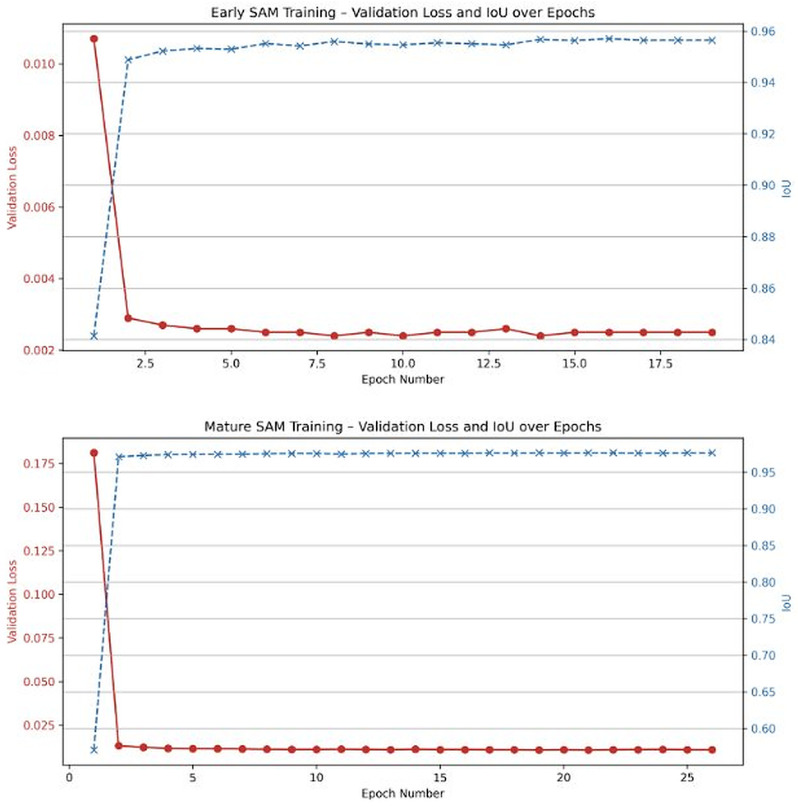
Validation loss and IoU across epochs for early- and late-stage SAM models. Red: validation loss (left axis); blue: IoU (right axis).

Training curves (Figure 7) show rapid convergence and stable validation loss for both models. The distinct convergence patterns reflect stage-specific segmentation characteristics and support the dual-model strategy over a single general-purpose model.

### 4.3. Pipeline Performance Across Developmental Stages

**Figure 8:**
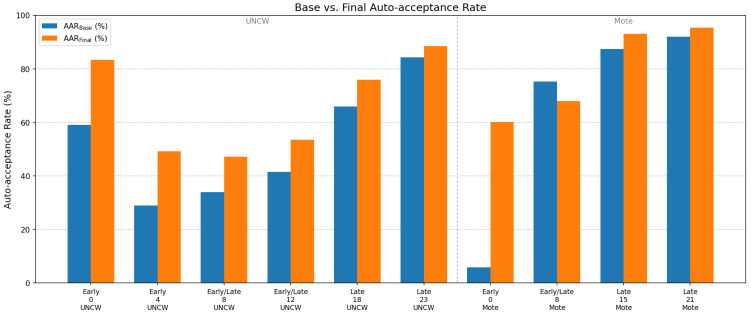
Auto-acceptance rate (AAR) improvement across developmental stages.

The baseline used YOLOv8 with a pretrained SAM ViT-H and bounding-box prompts only. The final pipeline incorporated YOLOv11, dual stage-specific fine-tuned SAM2-Hiera-Large models, and bounding box with four background point prompts. Both were evaluated on the UNCW 2024 and Mote 2024 datasets (Table 6, Figure 8).

The baseline performed reliably on late-stage recruits, achieving an AAR of 65–92% at later time points with median area ratios near unity (0.96–0.99), indicating slight but consistent underestimation. Early-stage performance was substantially weaker, with an AAR of 6–59% driven by overestimation from tissue transparency and illumination artifacts. This was most pronounced in the Mote dataset, where segmentation leakage into the corallite cup produced median area ratios of 1.04–1.21. Intermediate time points in the UNCW dataset (weeks 8–12), containing mixed stages, yielded a moderate AAR of 34–42%. Mote week 8, which visually resembled late-stage recruits, achieved a higher AAR (75%) but retained early-stage-like overestimation (median area ratio ≈ 1.03).

The final pipeline improved performance across most time points. Gains were modest for late-stage recruits (AAR +3–10%) but substantial for early-stage recruits, where AAR increased by 20–54% and median area ratios shifted from 1.04–1.21 to 0.96–1.00, reflecting effective suppression of overestimation. At intermediate time points, the AAR improved by 12–13% in UNCW but declined moderately at Mote week 8 (−7.35%), where the median area ratio increased marginally from 1.03 to 1.04.

### 4.4. Ablation: Stage-Inverted Processing

To verify the dual-branch design, we conducted a stage-inversion experiment: early-stage images were processed with the late-stage model and configuration (no background prompts), and late-stage images with the early-stage model and configuration (with background prompts).

**Figure 9:**
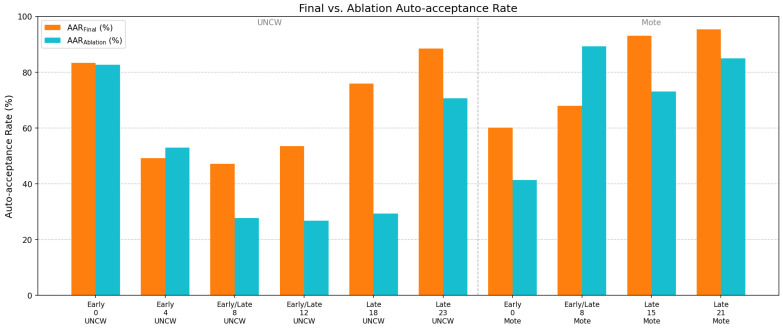
Visual comparison between the inverted and final auto-acceptance rate (AAR).

As shown in Table 7 and Figure 9, inversion degraded performance at 8 of 10 time points, with AAR reductions of up to 46.63% (UNCW week 18). The two exceptions both occurred at developmentally ambiguous time points: UNCW week 4 (early, ΔAAR =+3.79%) and Mote week 8 (early/late, ΔAAR = +21.38%). The Mote week 8 anomaly is notable—the inverted late-stage configuration outperformed the correct assignment, with median area ratios shifting from 1.036 to 1.003, suggesting these recruits were functionally closer to late-stage morphology despite their mixed-stage classification. These two exceptions aside, the consistent performance degradation under inversion confirms that the stage-specific model assignment is mechanistically justified. The dual-branch design performs correctly as designed for the majority of the developmental range (8 of 10 time points, with degradations up to 46.6%), but performance at morphologically ambiguous transition points motivates future work on finer-grained stage resolution.

Additional visualization and expanded descriptive statistics can be found in Supplementary Materials. Detailed descriptive statistics for the Area Ratio across all pipelines and time points are provided in Table S1, and additional visualizations are presented in Figures S1–S4 of the Supplementary Materials. Figure S1 summarizes AVG Area Ratio (±STD) for the Baseline, Final, and Ablation pipelines across developmental stages at both sites. Figure S2 provides a heatmap overview of AVG AR, Median AR, and AAR across pipelines and time points. Figures S3 and S4 display the full AR scatter plots and boxplots for the UNCW and Mote sites, respectively, confirming that the Final pipeline consistently compresses AR distributions toward unity with reduced bias and variability across most time points.

## 5. Discussion

This study presents a deep learning pipeline for automated coral recruit area measurement that integrates YOLO-based detection with SAM-based segmentation. Processing time was reduced from 2 to 7 min per image by manual tracing to approximately 3 to 5 s per image on T4 GPUs and around 3 s on A100 GPUs under typical conditions, a 24–140× speedup that removes a major analytical bottleneck and enables large-scale restoration studies previously constrained by labor.

The optimized pipeline achieved a mean IoU exceeding 95% and an overall AAR of 71.51%, where predicted-to-ground-truth area ratios fall within a ±5% tolerance of expert annotation. The remaining 28.49% fell outside the ±5% tolerance threshold; however, as shown in the Supplementary Materials, most predictions clustered closely around unity, indicating marginal deviations rather than systematic failures. Predicted segmentations remained biologically reasonable across both datasets with minimal correction required. This shifts the analytical burden from full manual tracing to lightweight quality control, preserving measurement reliability at substantially reduced effort.

A key strength is generalization across datasets collected under distinct experimental conditions. Despite differences in imaging protocols, substrate types, water treatments, coral species, and developmental stages, performance remained consistent across the UNCW and Mote datasets. This suggests the pipeline captures fundamental visual features of coral tissue rather than overfitting to site-specific artifacts, supporting deployment across multiple laboratories and restoration programs.

**Figure 10:**
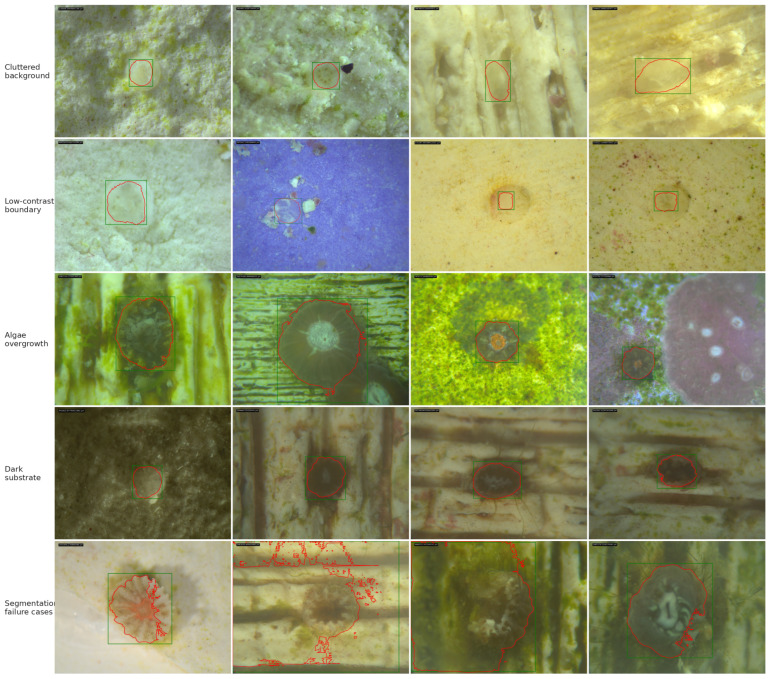
Representative challenging coral recruit microscopy images across developmental stages, including tissue transparency, weak contrast, skeletal cup interference, uneven illumination, symbiont-dominated morphology, and increasing structural complexity. Red contours show model-predicted segmentations and green boxes indicate detection bounding boxes. Visually plausible masks can still produce large area-ratio deviations due to biological ambiguity and imaging artifacts.

The hierarchical stage-specific architecture directly addresses the biological challenge of rapid morphological change during early development. Early-stage recruits, characterized by translucent tissue and poorly defined tissue–skeleton boundaries, caused over-segmentation in generic models; incorporating a specialized early-stage SAM with background point prompts effectively suppressed segmentation leakage. For late-stage recruits, where tissue boundaries are more distinct, the late-stage model achieved consistently strong performance with only slight underestimation. Together, these design choices extend reliable measurement across the full recruitment trajectory. As shown in Figure 10, representative challenging cases illustrate the practical performance limits imposed by early developmental morphology, symbiont contrast, and illumination effects.

Several limitations warrant consideration. Pipeline accuracy depends on reliable YOLO detections, and extreme imaging conditions or irregular growth patterns can degrade bounding-box localization and propagate errors downstream. SAM fine-tuning requires substantial computational resources, which may limit accessibility for groups without modern hardware. Future work could explore lightweight segmentation architectures or transfer learning strategies that reduce computational demands while preserving accuracy. The A–G developmental stage classification was derived through systematic expert review of hundreds of recruit images and two-researcher consensus, and retains an inherent element of subjectivity. Future work should explore automating stage assignment using quantitative criteria (e.g., size, shape, or symbiont acquisition metrics) and establishing formal inter-rater reliability (e.g., Cohen’s kappa) to improve reproducibility and reduce manual intervention.

Although the pipeline performed well across four reef-building coral species over 0–23 weeks, validation on a broader taxonomic range and longer temporal scales remains necessary to confirm generalizability. Future studies could quantify tissue–substrate boundary contrast and intensity gradients across developmental stages, which would allow direct measurement of the biological contribution to segmentation uncertainty and help disentangle biological from algorithmic performance limits. A fully factorial ablation isolating the independent contributions of YOLO version, SAM architecture, and stage-specific configuration was outside the scope of this deployment-driven study; systematic component-wise evaluation represents a valuable direction for future work.

Object detection architectures have evolved rapidly since YOLOv8 [31], with subsequent iterations introducing improvements in accuracy, inference speed, and robustness under challenging imaging conditions. YOLOv11 [32] improved detection under clutter and low-contrast boundaries, directly motivating its adoption in our pipeline for early-stage coral recruit detection. More recent architectures—including transformer-based detectors and the next generation of foundation segmentation models—further advance detection precision and boundary delineation; however, as these models were released during the final stages of this work and the field continues to evolve rapidly, systematic benchmarking within coral morphometric pipelines remains an open research direction that we prioritize for future work.

The modular pipeline design extends naturally beyond coral recruits—with modest modifications, similar workflows could support larval settlement monitoring in oysters, sponges, or algae. Future directions include real-time deployment in coral nurseries, volumetric measurement via 3D imaging, cloud-based interfaces for broader community access, and active or self-supervised learning to reduce annotation burden in long-term, multi-environment monitoring. By automating recruit measurement with high accuracy and efficiency, this pipeline enables more scalable and standardized restoration research. As reef restoration efforts expand globally, tools that reduce manual labor while maintaining biological rigor will be critical for timely conservation decisions. Consistent with this potential, the pipeline has been operational at multiple coral research facilities since March 2025, supporting routine monitoring beyond the validation setting and demonstrating robustness to cross-institutional variation in imaging conditions. All participating laboratories run the pipeline via Google Colab with GPU runtime, which provides accessible T4 or A100 GPUs without requiring local hardware infrastructure.

## 6. Conclusions

We present a deep learning pipeline that automates coral recruit detection and surface area measurement in microscopy images, reducing processing time by 24–140× relative to manual tracing while achieving a mean IoU exceeding 95% and an overall AAR of 71.51%. Stage-specific SAM models and background point prompts were critical for handling morphological differences between early- and late-stage recruits, and the system generalized consistently across species, datasets, and imaging conditions. Remaining limitations include computational demands for fine-tuning and the need for validation across a broader taxonomic range.

As reef restoration efforts scale globally, automated tools that reduce manual labor while maintaining biological rigor are increasingly necessary. The modular design of this pipeline makes it adaptable to related ecological imaging tasks, and future extensions—including real-time deployment and 3D volumetric measurement—could further expand its utility for large-scale coral monitoring.

## Figures and Tables

**Table 1:** Overview of coral growth projects used in the study.

Lab	Year	Species	Time Points Sampled	Approx Images Declining
UNCW	2023	PCLI	7 (every ∼4 weeks)	400 → 200
UNCW	2024	MCAV	7 (every ∼4 weeks)	400 → 200
Mote	2024	CNAT, PSTR	4 (every ∼8 weeks)	600 → 400

**Table 2:** Stage-specific SAM2 segmentation configurations.

Component	Early-Stage SAM2	Late-Stage SAM2
SAM architecture	SAM2-Hiera-Large	same
YOLO input	Bounding box + stage label	same
Bounding-box prompt	Yes	same
Background points	Four (box-edge midpoints)	None
Foreground points	None	same
Fine-tuned weights	Early-stage specific	Late-stage specific
Primary objective	Suppress cup over-segmentation	Preserve tissue boundaries
Typical tissue contrast	Low/indistinct	High/well-defined

**Table 3:** Comparison between the baseline (prototype) and final (optimized) YOLO–SAM pipeline configurations.

Component	Baseline (Prototype)	Final (Optimized)
YOLO version	YOLOv8	YOLOv11
YOLO output	Bounding box only	Bounding box + stage classification
SAM architecture	SAM ViT-H (pretrained)	SAM2-Hiera-Large
SAM fine-tuning	None	Dual stage-specific fine-tuned models
Stage-specific switching	No	Yes (early vs. late routing)
Bounding-box prompt	Yes	Yes
Background points	None	Four (box-edge midpoints, early only)
Segmentation strategy	Single SAM model	Stage-specific SAM2 models

**Table 4:** YOLOv11 detection performance on the test set (All = combined early + late; early/late = class-specific).

Class	Images/ Instances	Precision	Recall	mAP@50	mAP@50-95
All	417	0.972	0.986	0.993	0.911
Early	208	0.971	0.986	0.992	0.873
Late	209	0.973	0.986	0.994	0.948

**Table 5:** Loss and IoU metrics for Early and Late SAMs on the test set.

Model	Loss	IoU
Early SAM	0.0025	0.9598
Late SAM	0.0100	0.9760

**Table 6:** Performance comparison between baseline and final pipelines across datasets and developmental stages.

Dataset	Week	Stage	AAR_Base_	AAR_Final_	ΔAAR	MAR_Base_	MAR_Final_	Δ(|Bias|)
UNCW	0	Early	59.03	83.33	+24.30	1.037	0.989	+0.026
UNCW	4	Early	28.91	49.17	+20.26	1.038	0.964	+0.002
UNCW	8	Early/Late	33.96	47.19	+13.23	0.959	0.954	−0.005
UNCW	12	Early/Late	41.49	53.56	+12.07	0.965	0.955	−0.010
UNCW	18	Late	65.87	75.96	+10.09	0.967	0.970	+0.003
UNCW	23	Late	84.29	88.48	+4.19	0.979	0.980	+0.001
Mote	0	Early	5.86	60.13	+54.27	1.205	0.995	+0.200
Mote	8	Early/Late	75.28	67.93	−7.35	1.026	1.036	−0.010
Mote	15	Late	87.36	93.10	+5.74	0.991	0.999	+0.008
Mote	21	Late	92.00	95.29	+3.29	0.988	0.997	+0.009

AAR: auto-acceptance rate, percentage of predictions within 5% of ground-truth area. ΔAAR = AAR_Final_ − AAR_Base_; positive values indicate improved auto-acceptance in the final pipeline. MAR: median of $AR_{i}=A_{i}^{\mathrm{pred}}/A_{i}^{\mathrm{true}}$; values near 1 indicate low systematic bias. Δ(|Bias|): reduction in absolute deviation from unity, $|{\mathrm{MAR}}_{\mathrm{Base}}-1|\;-\;|{\mathrm{MAR}}_{\mathrm{Final}}-1|$; positive values indicate improved agreement with ground truth.

**Table 7:** Pipeline performance under inverted stage-model assignment, validating the dual-branch SAM design.

Dataset	Week	Stage	AAR_Final_	AAR_Ablation_	ΔAAR	MAR_Final_	MAR_Ablation_	Δ(|Bias|)
UNCW	0	Early	83.33	82.64	−0.69	0.989	1.013	−0.002
UNCW	4	Early	49.17	52.96	+3.79	0.964	0.995	+0.031
UNCW	8	Early/Late	47.19	27.72	−19.47	0.954	0.935	−0.019
UNCW	12	Early/Late	53.56	26.78	−26.78	0.955	0.938	−0.017
UNCW	18	Late	75.96	29.33	−46.63	0.970	0.945	−0.025
UNCW	23	Late	88.48	70.68	−17.80	0.980	0.961	−0.019
Mote	0	Early	60.13	41.37	−18.76	0.995	1.057	−0.052
Mote	8	Early/Late	67.93	89.31	+21.38	1.036	1.003	+0.033
Mote	15	Late	93.10	73.10	−20.00	0.999	0.966	−0.033
Mote	21	Late	95.29	84.94	−10.35	0.997	0.971	−0.026

AAR: auto-acceptance rate, percentage of predictions within 5% of ground-truth area. ΔAAR = AAR_Ablation_ − AAR_Final_; negative values confirm that correct stage assignment outperforms inverted assignment. MAR: median of $AR_{i}=A_{i}^{\mathrm{pred}}/A_{i}^{\mathrm{true}}$; values near 1 indicate low systematic bias. Δ(|Bias|) = $|{\mathrm{MAR}}_{\mathrm{Final}}-1|$ − $|{\mathrm{MAR}}_{\mathrm{Ablation}}-1|$; negative values indicate lower systematic bias in the final pipeline.

## Data Availability

Selected components of this project may be made available by the corresponding authors upon reasonable request, subject to institutional agreements and data-use policies.

## Supplementary Materials

The following supporting information can be downloaded at: https://www.mdpi.com/article/10.3390/s26082291/s1, Table S1: Descriptive statistics of Baseline, Final, and Ablation Pipeline area ratio across time points for UNCW and Mote sites; Figure S1: AVG Area Ratio (± STD) across developmental stages for UNCW (Weeks 0, 4, 8, 12, 18, 23) and Mote (Weeks 0, 8, 15, 21) sites: (a) Baseline vs. Final pipeline; (b) Final vs. Ablation pipeline; Figure S2: Heatmap summary of pipeline performance across time points for UNCW (Weeks 0, 4, 8, 12, 18, 23) and Mote (Weeks 0, 8, 15, 21): (a) AVG Area Ratio; (b) Median Area Ratio; (c) Auto-Accept Rate (%); Figure S3: Area Ratio distributions across all time points for the UNCW site (Weeks 0, 4, 8, 12, 18, 23) for Baseline, Final, and Ablation pipelines; Figure S4: Area Ratio distributions across all time points for the Mote site (Weeks 0, 8, 15, 21) for Baseline, Final, and Ablation pipelines.

## Abbreviations

The following abbreviations are used in this manuscript:

YOLO	You Only Look Once
SAM	Segment Anything Model
AAR	Auto-acceptance rate
IoU	Intersection over Union
PCLI	Pseudodiploria clivosa
MCAV	Montastraea cavernosa
CNAT	Colpophyllia natans
PSTR	Pseudodiploria strigosa
Mote	Mote Marine Laboratory and Aquarium

## References

[1] Eddy T.D., Lam V.W., Reygondeau G., Cisneros-Montemayor A.M., Greer K., Palomares M.L.D., Bruno J.F., Ota Y., Cheung W.W. (2021). Global decline in capacity of coral reefs to provide ecosystem services. One Earth.

[2] Moberg F., Folke C. (1999). Ecological goods and services of coral reef ecosystems. Ecol. Econ..

[3] Spalding M., Burke L., Wood S.A., Ashpole J., Hutchison J., zu Ermgassen P. (2017). Mapping the global value and distribution of coral reef tourism. Mar. Policy.

[4] Bruno J.F., Valdivia A. (2016). Coral reef degradation is not correlated with local human population density. Sci. Rep..

[5] Field C.B., Barros V.R., Dokken D.J., Mach K.J., Mastrandrea M.D., Bilir T.E., Chatterjee M., Ebi K.L., Estrada Y.O., Genova R.C. (2014). Climate Change 2014: Impacts, Adaptation, and Vulnerability. Part A: Global and Sectoral Aspects.

[6] Hughes T.P., Kerry J.T., Álvarez-Noriega M., Álvarez-Romero J.G., Anderson K.D., Baird A.H., Babcock R.C., Beger M., Bellwood D.R., Berkelmans R. (2017). Global warming and recurrent mass bleaching of corals. Nature.

[7] Hoegh-Guldberg O., Anthony K., Berkelmans R., Dove S., Fabricius K., Lough J., Marshall P., van Oppen M., Negri A., Willis B. (2007). Vulnerability of Reef-Building Corals on the Great Barrier Reef to Climate Change.

[8] Voolstra C.R., Peixoto R.S., Ferrier-Pagès C. (2023). Mitigating the ecological collapse of coral reef ecosystems. EMBO Rep..

[9] Edmunds P.J. (2023). Coral recruitment: Patterns and processes determining the dynamics of coral populations. Biol. Rev. Camb. Philos. Soc..

[10] Levenstein M.A., Marhaver K.L., Quinlan Z.A., Tholen H.M., Tichy L., Yus J., Lightcap I., Wegley Kelly L., Juarez G., Vermeij M.J.A. (2022). Composite Substrates Reveal Inorganic Material Cues for Coral Larval Settlement. ACS Sustain. Chem. Eng..

[11] Craggs J., Guest J.R., Davis M., Simmons J., Dashti E., Sweet M. (2017). Inducing broadcast coral spawning ex situ: Closed system mesocosm design and husbandry protocol. Ecol. Evol..

[12] Chamberland V.F., Vermeij M.J., Brittsan M., Carl M., Schick M., Snowden S., Schrier A., Petersen D. (2015). Restoration of critically endangered elkhorn coral (*Acropora palmata*) populations using larvae reared from wild-caught gametes. Glob. Ecol. Conserv..

[13] Khodzori F.A., Roger N.A.A., Nor’ashikin A.Z., Azseri A., Misi L.L., Mazni M.A., Hisham H.K., Shah M.D., Chong W.S., Faudzi N.M., Shah M.D., Mazlan N., Raehanah Muhamad Shaleh S. (2024). Coral Aquaculture: A Review of In Situ and Ex Situ Culture Systems, Conditions, Applications, and Challenges. Essentials of Aquaculture Practices.

[14] Zweifler A., Akkaynak D., Mass T., Treibitz T. (2017). In situ Analysis of Coral Recruits Using Fluorescence Imaging. Front. Mar. Sci..

[15] Bahr K.D., Tran T., Jury C.P., Toonen R.J. (2020). Abundance, size, and survival of recruits of the reef coral Pocillopora acuta under ocean warming and acidification. PLoS ONE.

[16] Koch H.R., Wallace B., DeMerlis A., Clark A.S., Nowicki R.J. (2021). 3D Scanning as a Tool to Measure Growth Rates of Live Coral Microfragments Used for Coral Reef Restoration. ProQuest.

[17] O’Neil K.L., Serafin R.M., Patterson J.T., Craggs J.R.K. (2021). Repeated ex situ spawning in two highly disease susceptible corals in the family Meandrinidae. Front. Mar. Sci..

[18] Macadam A., Nowell C.J., Quigley K. (2021). Machine Learning for the Fast and Accurate Assessment of Fitness in Coral Early Life History. Remote Sens..

[19] Wong Y.K., Zheng Z., Zhang M., Suggett D., Yeung S.K. (2024). CoralSCOP-LAT: Labeling and Analyzing Tool for Coral Reef Images with Dense Mask. arXiv.

[20] Beijbom O., Edmunds P.J., Roelfsema C., Smith J., Kline D.I., Neal B.P., Dunlap M.J., Moriarty V., Fan T.Y., Tan C.J. (2015). Towards Automated Annotation of Benthic Survey Images: Variability of Human Experts and Operational Modes of Automation. PLoS ONE.

[21] Richards B.L., Beijbom O., Campbell M.D., Clarke M.E., Cutter G., Dawkins M., Edington D., Hart D.R., Hill M.C., Hoogs A. (2019). Automated Analysis of Underwater Imagery: Accomplishments, Products, and Vision.

[22] González-Rivero M., Beijbom O., Rodriguez-Ramirez A., Bryant D.E.P., Ganase A., Gonzalez-Marrero Y., Herrera-Reveles A., Kennedy E.V., Kim C.J.S., Lopez-Marcano S. (2020). Monitoring of Coral Reefs Using Artificial Intelligence: A Feasible and Cost-Effective Approach. Remote Sens..

[23] Xu M., Su J., Liu Y. (2023). AquaSAM: Underwater Image Foreground Segmentation. arXiv.

[24] Chowdhury A., Jahan M., Kaisar S., Khoda M.E., Rajin S., Naha R. (2024). Coral Reef Surveillance with Machine Learning: A Review of Datasets, Techniques, and Challenges. Electronics.

[25] LeCun Y., Bengio Y., Hinton G. (2015). Deep learning. Nature.

[26] Guo Y., Liu Y., Oerlemans A., Lao S., Wu S., Lew M.S. (2016). Deep learning for visual understanding: A review. Neurocomputing.

[27] Pandey S., Chen K.F., Dam E.B. (2023). Comprehensive Multimodal Segmentation in Medical Imaging: Combining YOLOv8 with SAM and HQ-SAM Models. arXiv.

[28] Perez L., Wang J. (2017). The Effectiveness of Data Augmentation in Image Classification using Deep Learning. arXiv.

[29] Shorten C., Khoshgoftaar T.M. (2019). A survey on Image Data Augmentation for Deep Learning. J. Big Data.

[30] Redmon J., Divvala S., Girshick R., Farhadi A. (2016). You Only Look Once: Unified, Real-Time Object Detection. arXiv.

[31] Jocher G., Chaurasia A., Qiu J. (2023). Ultralytics YOLOv8. https://github.com/ultralytics/ultralytics.

[32] Jocher G., Qiu J. (2024). Ultralytics YOLO11. https://docs.ultralytics.com/models/yolo11/.

[33] Kirillov A., Mintun E., Ravi N., Mao H., Rolland C., Gustafson L., Xiao T., Whitehead S., Berg A.C., Lo W.Y. (2023). Segment Anything. Proceedings of the IEEE/CVF International Conference on Computer Vision (ICCV).

[34] Ravi N., Gabeur V., Hu Y.T., Hu R., Ryali C., Ma T., Khedr H., Rädle R., Rolland C., Gustafson L. (2024). SAM 2: Segment Anything in Images and Videos. arXiv.

