# Supplementary material for: Hierarchical YOLO-SAM: A Scalable Pipeline for Automated Segmentation and Morphometric Tracking of Coral Recruits in Time-Series Microscopy
Source: Sensors (Basel). 2026 Apr 8;26(8):2291. doi: 10.3390/s26082291 (PMC13119570; doi:10.3390/s26082291)
Supplement: Supplementary file 1 [file sensors-26-02291-s001.zip › sensors-4195942-supplementary.pdf]

sensors

MDPI

Article

Hierarchical YOLO-SAM: A Scalable Pipeline for Automated Segmentation and Morphometric Tracking of Coral Recruits in Time-Series Microscopy

Richard S. Zhao [1,*], Cuixian Chen [2,*], Meg Van Horn [3] and Nicole D. Fogarty [3]

[1] Department of Computer Science, University of North Carolina at Chapel Hill, Chapel Hill, NC 27599, USA
[2] Department of Mathematics and Statistics, University of North Carolina Wilmington, Wilmington, NC 28403, USA
[3] Department of Biology and Marine Biology, Center for Marine Science, University of North Carolina Wilmington, Wilmington, NC 28403, USA; meg.vanhorn98@gmail.com (M.V.H.); fogartyn@uncw.edu (N.D.F.)
* Correspondence: richardszhao@gmail.com (R.S.Z.); chenc@uncw.edu (C.C.)

Supplementary Materials

The appendix serves solely as supplementary material to Sections 4.3 and 4.4. All primary analysis and detailed discussion are presented in these two sections. The materials provided in the appendix are intended only for additional visualization and expanded descriptive statistics. Readers are encouraged to refer to Sections 4.3 and 4.4 for the full interpretation and discussion of results.

1. Expanded descriptive statistics and additional visualization

Table S1 and Figure S1 present descriptive statistics for the Area Ratio (AR) under the Baseline (Panel A), Final (Panel B), and Ablation (Panel C) pipelines across all time points at the UNCW (Weeks 0–23) and Mote (Weeks 0–21) sites. Comparing Panels A and B, the Final pipeline consistently reduced mean AR toward 1.0 and narrowed STD at most time points. At UNCW, the largest reduction in absolute bias from AR = 1 for UNCW occurred at Week 12 (0.107), reflecting a strong correction of baseline overestimation. The largest reduction in absolute bias for Mote occurred at Week 0 (0.204), indicating a substantial correction of baseline overestimation. Comparing Panels B and C, the Final and Ablation pipelines produced slightly different patterns of mean AR and STD across both sites. Overall, mean AR toward 1.0 improves over Ablation mainly on Mote, but not consistently on UNCW.

Check for updates

Academic Editors: Stefano Berretti, Jean-Baptiste Thomas, Baptiste Magnier and Khizar Hayat

Received: 24 February 2026
Revised: 31 March 2026
Accepted: 31 March 2026
Published: 8 April 2026

Copyright: © 2026 by the authors. Licensee MDPI, Basel, Switzerland. This article is an open access article distributed under the terms and conditions of the Creative Commons Attribution (CC BY) license.

Table S1. Descriptive Statistics of baseline, final, and ablation pipeline area ratio across time points for UNCW and Mote sites

UNCW							Mote				
**Week**	**0**	**4**	**8**	**12**	**18**	**23**	**Week**	**0**	**8**	**15**	**21**
Panel A: Baseline Pipeline											
AVG AR	1.052	1.105	1.033	1.154	1.369	1.020	AVG AR	1.214	1.029	1.014	1.000
STD	0.084	0.395	0.353	0.740	1.491	0.323	STD	0.138	0.084	0.331	0.154
25%	1.011	0.955	0.926	0.938	0.954	0.971	25%	1.140	1.009	0.977	0.979
50%	1.037	1.038	0.959	0.965	0.967	0.979	50%	1.205	1.026	0.991	0.988
75%	1.073	1.148	1.025	1.041	0.978	0.992	75%	1.283	1.048	1.004	0.996
Min.	0.583	0.000	0.749	0.740	0.794	0.000	Min.	0.000	0.000	0.213	0.839
Max.	1.543	5.201	5.314	6.849	13.106	4.284	Max.	1.748	1.340	5.894	3.766
AAR (%)	59.028	28.910	33.955	41.494	65.865	84.293	AAR (%)	5.863	75.278	87.356	92.000
Panel B: Final Pipeline											
AVG AR	0.992	0.961	0.963	1.047	1.264	1.012	AVG AR	1.010	1.038	1.014	1.008
STD	0.043	0.102	0.107	0.441	1.236	0.255	STD	0.084	0.041	0.237	0.126
25%	0.972	0.906	0.923	0.931	0.959	0.973	25%	0.961	1.019	0.990	0.991
50%	0.989	0.964	0.954	0.955	0.970	0.980	50%	0.995	1.036	0.999	0.997
75%	1.013	1.003	0.982	0.983	0.980	0.987	75%	1.042	1.055	1.010	1.006
Min.	0.806	0.524	0.749	0.820	0.809	0.703	Min.	0.748	0.750	0.830	0.911
Max.	1.189	1.627	1.800	4.671	11.482	3.202	Max.	1.454	1.238	5.876	3.533
AAR (%)	83.333	49.173	47.191	53.556	75.962	88.482	AAR (%)	60.134	67.929	93.103	95.294
Panel C: Ablation Pipeline											
AVG AR	1.016	0.994	0.955	1.036	1.245	0.993	AVG AR	1.074	1.006	0.967	0.982
STD	0.043	0.114	0.116	0.456	1.290	0.253	STD	0.101	0.042	0.065	0.144
25%	0.992	0.939	0.901	0.914	0.933	0.950	25%	1.009	0.987	0.951	0.960
50%	1.013	0.995	0.935	0.938	0.945	0.961	50%	1.057	1.003	0.966	0.971
75%	1.035	1.041	0.993	0.988	0.958	0.971	75%	1.128	1.024	0.979	0.984
Min.	0.849	0.621	0.681	0.789	0.808	0.651	Min.	0.717	0.660	0.001	0.805
Max.	1.247	1.711	1.802	4.838	12.012	3.125	Max.	1.910	1.288	1.544	3.839
AAR (%)	82.639	52.955	27.715	26.778	29.327	70.681	AAR (%)	41.374	89.310	73.103	84.941
# Images	432	422	268	241	208	191	# Images	597	449	435	425

AR: area ratio. $AR_i = A_i^{\text{pred}} / A_i^{\text{true}}$; values $=1$, >1, and <1 indicate perfect agreement, over-, and underestimation, respectively. AAR: auto-acceptance rate, percentage of predictions within 5% of ground-truth area.

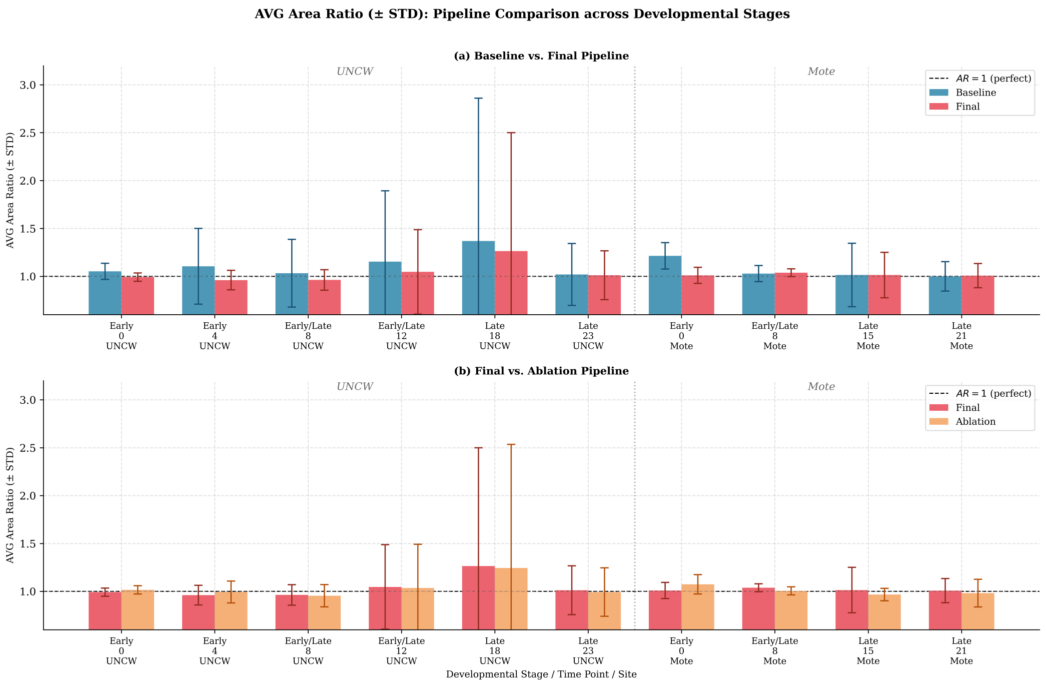

Figure S1. AVG Area Ratio (± STD) across developmental stages for UNCW (Weeks 0, 4, 8, 12, 18, 23) and Mote (Weeks 0, 8, 15, 21) sites. (a) Baseline vs. Final pipeline; (b) Final vs. Ablation pipeline. Dashed line at $AR = 1$ denotes perfect agreement. All panels share the same y-axis scale. Error bars represent one standard deviation.

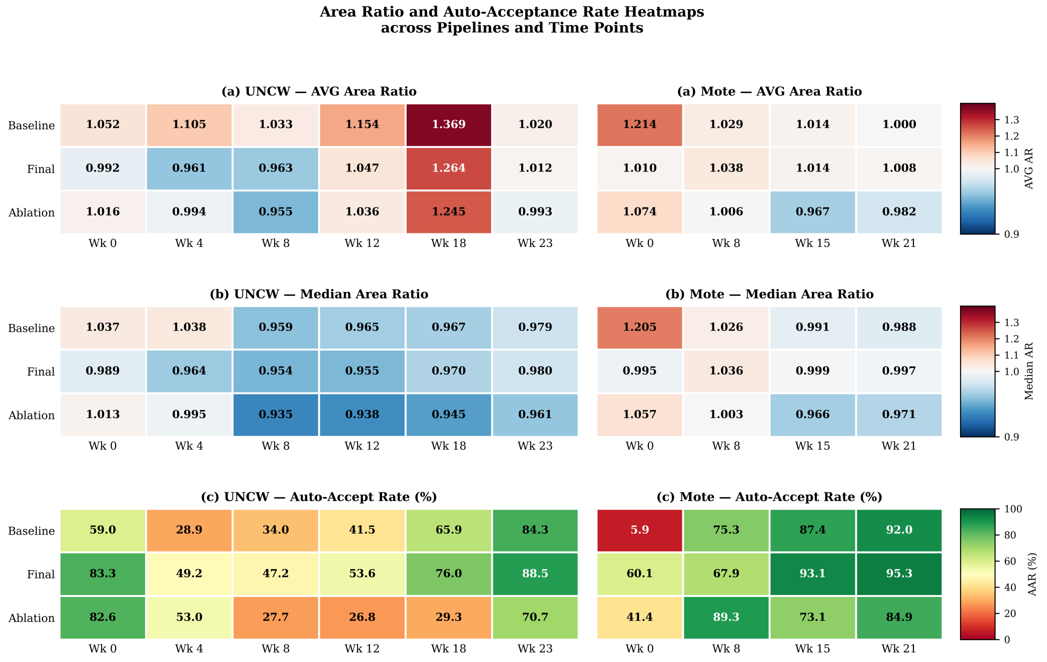

Figure S2. Heatmap summary of pipeline performance across time points for UNCW (Weeks 0, 4, 8, 12, 18, 23) and Mote (Weeks 0, 8, 15, 21). (a) AVG Area Ratio; (b) Median Area Ratio; (c) Auto-Accept Rate (%). In (a) and (b), color diverges from white ($AR = 1$, perfect agreement) toward red (overestimation) and blue (underestimation). In (c), color ranges from red (low AAR) to green (high AAR).

Figure S2 visualizes mean AR, median AR, and AAR across pipelines and time points, highlighting patterns of overestimation and underestimation. The Final pipeline shifts AR values closer to 1.0 relative to the Baseline, while the Ablation shows similar AR patterns at

https://doi.org/10.3390/s26082291

most time points. Panel (c) further illustrates improved reliability of the Final pipeline in AAR. Full interpretation and discussion of results can be found in Sections 4.3 and 4.4.

Figure S3 and Figure S4 display the AR scatter plots and boxplots for the Baseline, Final and Ablation pipeline at the UNCW (Weeks 0, 4, 8, 12, 18, 23) and Mote (Weeks 0, 8, 15, 21) sites, respectively. The final pipeline compresses AR distributions toward $AR = 1$, with denser scatter and narrower boxplots than the baseline at most time points. The largest improvements occur at UNCW Weeks 12, while Week 18 remains the most variable. At Mote, the elevated baseline bias at Week 0 is largely corrected, and Weeks 15 and 21 show near-ideal distributions; Week 8 is the only minor exception. Overall, the final pipeline achieves reduced bias and variability and improved reliability across most time points.

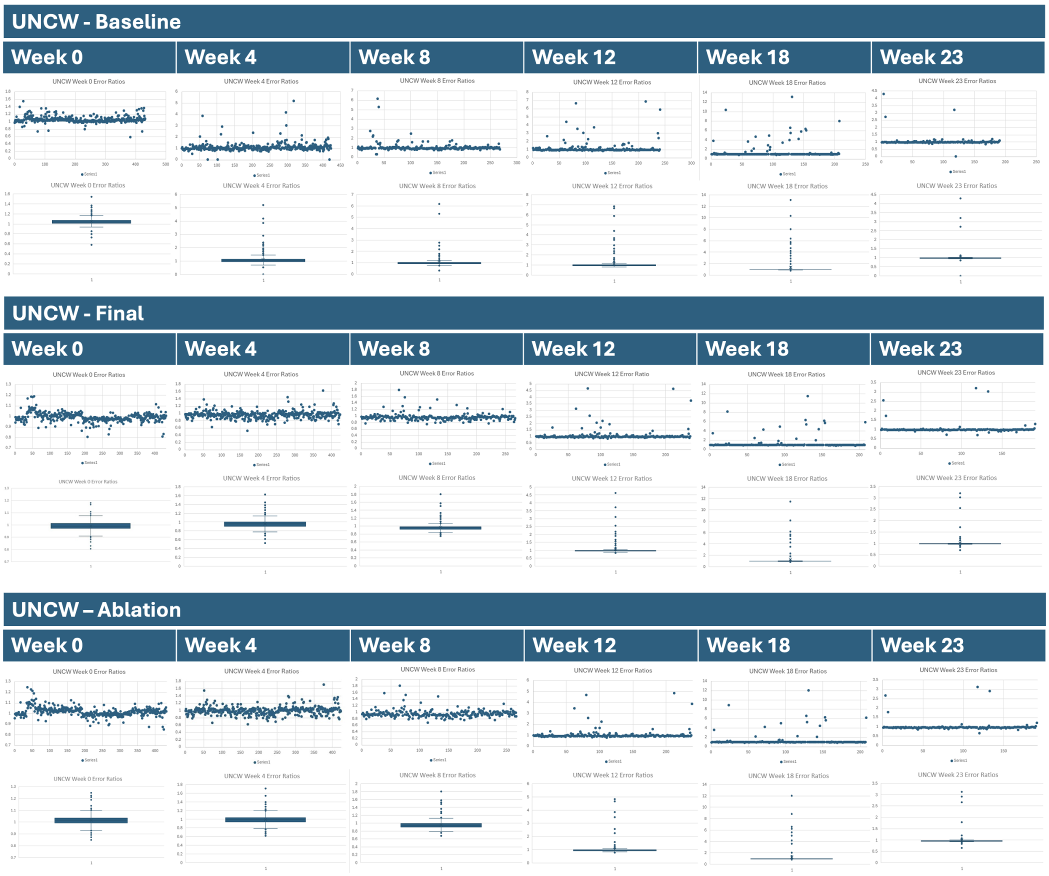

Figure S3. Area Ratio (AR) distributions across all time points for the UNCW site (Weeks 0, 4, 8, 12, 18, 23). Rows show the Baseline (top), Final (middle), and Ablation (bottom) pipelines. Within each panel, scatter plots (top) and boxplots (bottom) are shown per time point. See Figure SS4 for Mote site results.

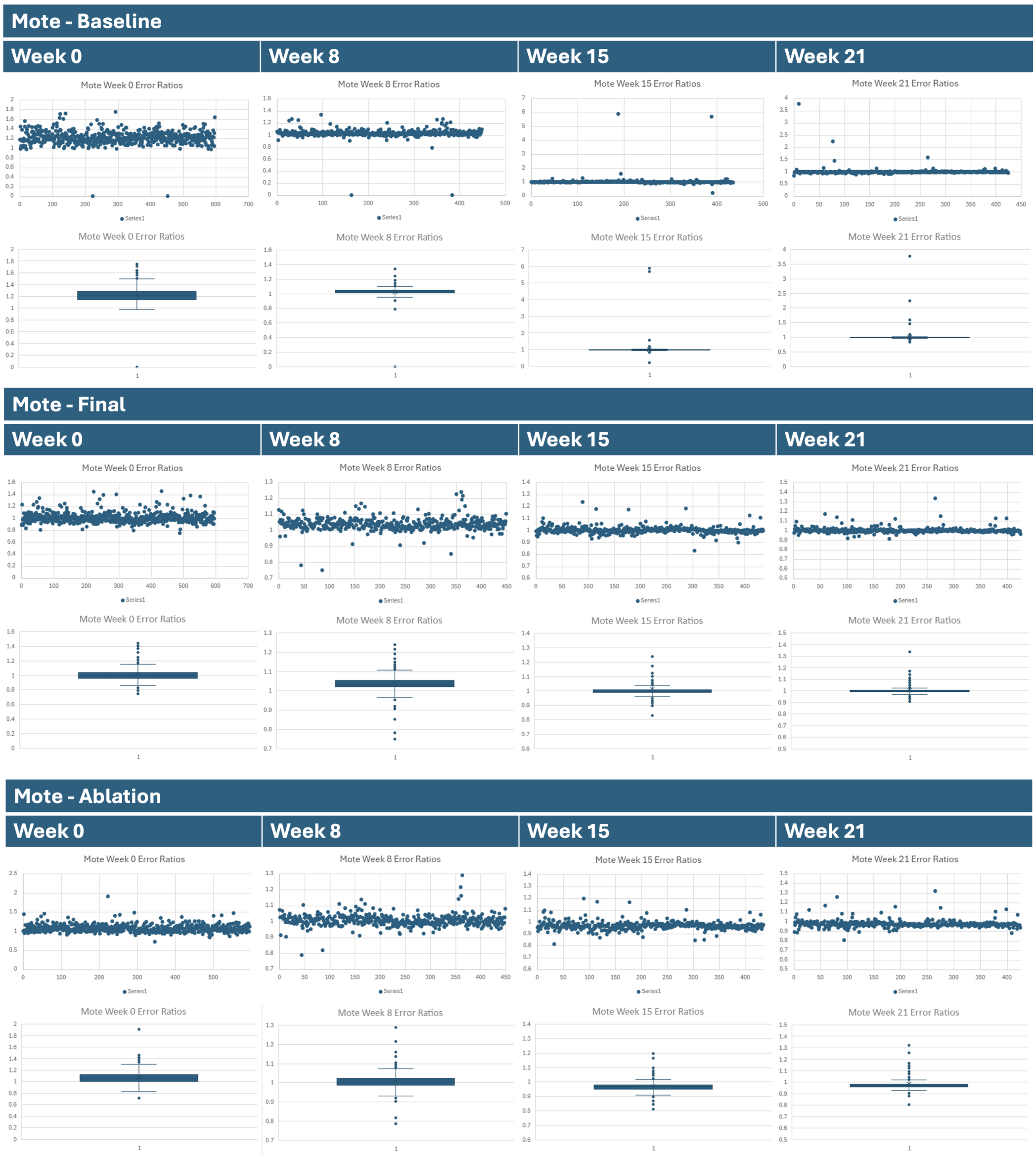

Figure S4. Area Ratio (AR) distributions across all time points for the Mote site (Weeks 0, 8, 15, 21). Rows show the Baseline (top), Final (middle), and Ablation (bottom) pipelines. Within each panel, scatter plots (top) and boxplots (bottom) are shown per time point. See Figure S3 for UNCW site results.

https://doi.org/10.3390/s26082291
